# Mitochondrial metabolism and the control of vascular smooth muscle cell proliferation

**DOI:** 10.3389/fcell.2014.00072

**Published:** 2014-12-15

**Authors:** Mario Chiong, Benjamín Cartes-Saavedra, Ignacio Norambuena-Soto, David Mondaca-Ruff, Pablo E. Morales, Marina García-Miguel, Rosemarie Mellado

**Affiliations:** ^1^Faculty of Chemical and Pharmaceutical Sciences, Advanced Center for Chronic Diseases, University of ChileSantiago, Chile; ^2^Faculty of Chemistry, Pontifical Catholic University of ChileSantiago, Chile

**Keywords:** vascular smooth muscle cell, proliferation, mitofusin-2, mitochondrial metabolism, mitochondrial dynamics

## Abstract

Differentiation and dedifferentiation of vascular smooth muscle cells (VSMCs) are essential processes of vascular development. VSMC have biosynthetic, proliferative, and contractile roles in the vessel wall. Alterations in the differentiated state of the VSMC play a critical role in the pathogenesis of a variety of cardiovascular diseases, including atherosclerosis, hypertension, and vascular stenosis. This review provides an overview of the current state of knowledge of molecular mechanisms involved in the control of VSMC proliferation, with particular focus on mitochondrial metabolism. Mitochondrial activity can be controlled by regulating mitochondrial dynamics, i.e., mitochondrial fusion and fission, and by regulating mitochondrial calcium handling through the interaction with the endoplasmic reticulum (ER). Alterations in both VSMC proliferation and mitochondrial function can be triggered by dysregulation of mitofusin-2, a small GTPase associated with mitochondrial fusion and mitochondrial–ER interaction. Several lines of evidence highlight the relevance of mitochondrial metabolism in the control of VSMC proliferation, indicating a new area to be explored in the treatment of vascular diseases.

## Introduction

Vascular smooth muscle cells (VSMCs) are the main component of the artery's medial layer. These cells undergo contraction and thereby regulate blood vessel tone and consequently blood flow and pressure. VSMC contraction depends on the interaction between smooth muscle α-actin, β-myosin heavy chain, h-caldesmon, and calponin (Rzucidlo et al., 2007; Cecchettini et al., 2011). VSMCs also possess important secretory properties that ensure synthesis and repair of extracellular matrix components and regulate the structure of the vascular wall (Cecchettini et al., 2011). Normal VSMCs are not terminally differentiated cells with very low rates of proliferation and secretion (Rzucidlo et al., 2007; Cecchettini et al., 2011). Changes in the VSMC phenotype have been extensively described in the development and progression of atherosclerosis, hypertension, and neointimal formation (Campbell and Campbell, 1985; Rzucidlo et al., 2007; Cecchettini et al., 2011). This phenotypic switching includes altered expression of contractile proteins, increased matrix production, expression of inflammatory cytokines, and production of proteases (Campbell and Campbell, 1985). The capacity for contraction, proliferation, migration, and secretion in VSMCs are affected by a wide range of factors, including mechanical forces, contractile agonists such as angiotensin II, extracellular matrix, reactive oxygen species (ROS), endothelial–VSMC interactions, platelet derived growth factor (PDGF), transforming growth factor-β1, hypoxia, and many other growth factors (Campbell and Campbell, 1985; Cecchettini et al., 2011). As a result, VSMCs constitute basic structural and functional elements in the artery wall and their malfunction leads to vascular disease. Recently, VSMC mitochondrial metabolism has been raised as part of novel mechanisms involved in the complex regulation of the VSMC phenotype, especially involving VSMC proliferation. In this review, we will examine the evidence that supports this hypothesis.

## Regulation of mitochondrial metabolism

Mitochondria have been considered the energy powerhouses in all eukaryotic cells (Scheffler, 2001). They are particularly abundant in muscle cells and most of the energy needed for muscle contraction is provided by mitochondrial metabolism (Kuznetsov et al., 2009). Mitochondria has four compartments: the outer mitochondrial membrane (OMM), the intermembrane space (IMS), the inner mitochondrial membrane (IMM), and the mitochondrial matrix (Scheffler, 2001). The IMM is particularly dense and enriched in a variety of membrane proteins, including the mitochondrial respiratory complexes and the ATP synthase (Scheffler, 2001). The driving energy for ATP synthesis comes from the generation of a proton motive force by the electron transport chain in the IMM. This gradient of concentration and charge constitutes the intermembrane mitochondrial potential (Δψm) and is maintained by the oxidation of reduced substrates generated mainly by the oxidation of metabolites in the Krebs cycle and β-oxidation of fatty acids (Scheffler, 2001; Kuzmicic et al., 2011).

VSMCs exhibit unusually high rates of glucose metabolization and lactate production under normal, well-oxygenated conditions (Butler and Siegman, 1985). Under resting conditions, the rate of oxygen consumption and lactate production are often almost equal on a molar basis, resulting in approximately 30% of the ATP supply coming from mitochondria, but at least 90% of the flux through glycolysis resulting in lactate production (Paul, 1983). In spite of the low contribution of mitochondria to the VSMC bioenergetics, mitochondrial dysfunction, and particularly mitochondrial DNA damage, has recently been associated to atherosclerosis (Yu and Bennett, 2014). Recent studies show that during VSMC phenotypic switching, mitochondria decreases glucose oxidation and increases fatty acid oxidation (Salabei and Hill, 2013). This suggests that VSMC redirects the use of glucose or its metabolites to pathways that support the biosynthesis of DNA and other molecules required for VSMC proliferation. This process is similar to the Warburg effect present in cancer cells (Vander Heiden et al., 2009).

Mitochondria were originally thought as discrete, isolated entities scattered in the cytoplasm. However, this vision has changed to a dynamic network model in which mitochondria are in close communication with the endoplasmic reticulum (ER) and the entire morphology of the organelle is controlled by coordinated fusion and fission events. The appropriate balance of these processes is essential to maintain mitochondrial stability and function (Kuzmicic et al., 2011; Parra et al., 2011).

## Mitochondrial dynamics and metabolism

**Figure 1:**
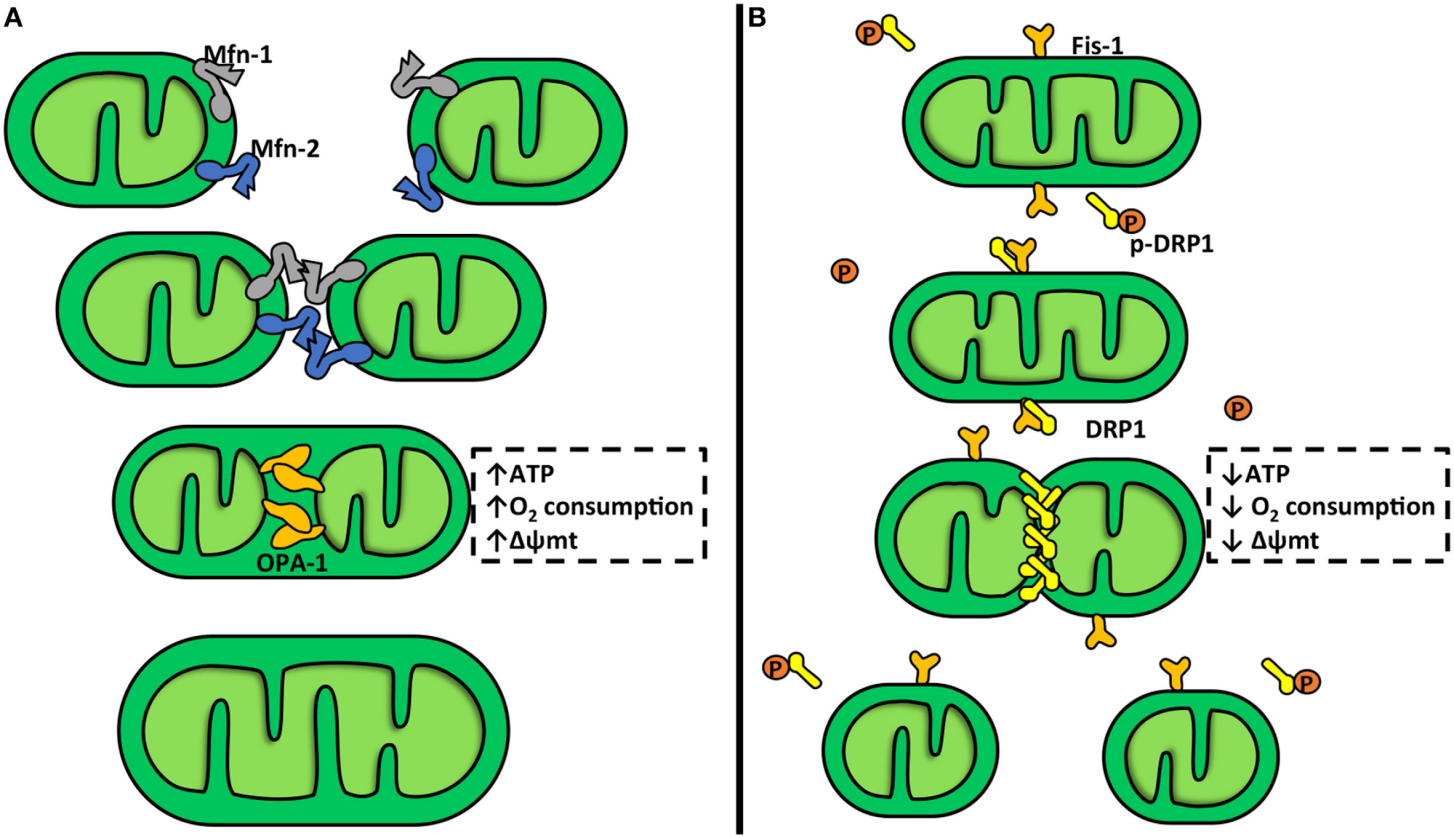
**Mitochondrial dynamics**. **(A)** Mitochondrial fusion. This is a two-step process which involves three different proteins: mitofusin-1 and 2 (Mfn-1 and Mfn-2) and optic atrophy protein-1 (OPA-1). Mfn-1 and Mfn-2 are transmembrane GTPases embedded in the outer mitochondrial membrane (OMM). The C-terminal coiled-coil region of Mfn-1 and Mfn-2 mediates tethering between mitochondria through homo- or heterotypic complexes formed between adjacent mitochondria. This interaction mediates OMM fusion. OPA-1 is a dynamin-related protein localized in the inner mitochondrial membrane (IMM), facing the intermembrane space. OPA-1 participates in the attachment and fusion of IMM. Mitochondrial fusion is associated with an increase in the mitochondrial potential (Δψm), oxygen consumption, and ATP production. **(B)** Mitochondrial fission. In this process participates dynamin-related protein-1 (DRP-1) and fission protein-1 (FIS-1). DRP-1 is a large GTPase found soluble in the cytosol of cells from where it shuttles onto and off mitochondria. DRP-1 assembles into spirals at division sites around the OMM to drive the fission process. In yeast, the mechanism for recruitment of DRP-1 to the mitochondria requires FIS-1, a tetratricopeptide domain protein anchored into and evenly coating the entire OMM. DRP-1 activity is inhibited by a protein kinase A (PKA) phosphorylation. Mitochondrial fission is associated with a Δψm, oxygen consumption, and ATP production decrease.

Mitochondrial fusion stimulates the assembly of individual mitochondria that combine their membranes (Figure 1A). This process is controlled by mitofusins (Mfn) 1 and 2 and OPA-1, a member of the dynamin family of mechanoenzymes (Parra et al., 2011). Mfn-1 and Mfn-2 are two evolutionarily conserved GTPase proteins that attach to the OMM via a bipartite, N-terminal transmembrane domain with their C-terminal coiled-coil region inserting into the cytosol. During mitochondrial fusion, Mfn-1 and Mfn-2 form hetero-oligomers that hydrolyze GTP and promote rearrangement of mitochondrial membranes (Carlucci et al., 2008). Mitochondrial fusion is a two-step process, where the IMM and OMM fuse by separate events (Malka et al., 2005; Zorzano et al., 2010), which can be explained by the mitochondrial sub-localization of Mfn-1, Mfn-2, and OPA-1. Mfn-1 and Mfn-2 are located in the OMM, where they can regulate the OMM fusion, and OPA-1 is located in the IMM, where it can regulate the IMM fusion (Zorzano et al., 2010). Mitochondrial fission entails fragmentation of tubular interconnected mitochondria into several smaller individual organelles (Figure 1B). The OMM protein FIS-1 and the GTPase dynamin related protein-1 (DRP-1) are the main elements of the mitochondrial fission machinery (Parra et al., 2011). Down regulation or inhibition of DRP-1 or FIS-1 severely inhibits mitochondrial fission and generates an extended mitochondrial membranous network (James et al., 2003; Cerveny et al., 2007). Furthermore, DRP-1 knock-out mice show that DRP-1 not only affects mitochondrial fission but also affects cell proliferation generating non-viable embryos (Wakabayashi et al., 2009). Ishihara et al. also showed that neural specific DRP-1 knock-out mice dies shortly after birth as a result of brain hypoplasia (Ishihara et al., 2009). This process is accompanied with an imbalance between mitochondrial fusion and fission, and deregulation of normal cytochrome c release and caspase activation during apoptosis (Ishihara et al., 2009). DRP-1 possesses an N-terminal GTPase domain and a C-terminal GTPase effector domain, which is involved in intramolecular and intermolecular interactions and modulates the GTPase activity. Cytosolic DRP-1 is recruited to the mitochondrial surface into the fission foci by adaptor proteins, including FIS-1 (Hoppins, 2014; Mishra and Chan, 2014; Nasrallah and Horvath, 2014). FIS-1 is an integral protein of the OMM and contains a cytosolic hydrophobic tetratricopeptide repeat (TPR). The TPR is thought to interact with DRP-1 (Carlucci et al., 2008; Hoppins, 2014). At fission sites, DRP-1 forms spiral chains around membrane constriction sites. GTP hydrolysis provides energy to generate the mechanical force required for fission (Carlucci et al., 2008). There are other adaptors that can recruit DRP-1 to the mitochondria membrane, i.e., the mitochondrial fission factor (MFF) and mitochondrial dynamic proteins 49 and 51 kDa (MiD49 and MiD51). These proteins can trigger mitochondrial fission through a FIS-1-independent process (Palmer et al., 2013).

Mitochondrial fission occurs preferentially at ER contact sites. Inverted formin 2 (INF2), a protein involved in actin polymerization and depolymerization located at the ER, has a direct effect on mitochondrial fission. INF2 facilitates both the interaction of DRP-1 with mitochondria and the fission process in an actin-dependent manner. Actin filaments appeared to accumulate between mitochondria and INF2-enriched ER membranes at constriction sites. Thus, INF2-induced actin filaments may drive initial mitochondrial constriction, which allows DRP-1-driven secondary constriction (Korobova et al., 2013).

Several lines of evidence have shown that the machinery that governs mitochondrial dynamics also participates in the temporal regulation of metabolism and cell death (Kuzmicic et al., 2011; Parra et al., 2011). The dynamic shifts in morphology coincide with a number of physiological events, such as transitions between different respiratory states and cristae remodeling during apoptosis (Parra et al., 2011). Aberrations in cristae morphology are accompanied by changes in metabolism (Parra et al., 2011; Nasrallah and Horvath, 2014), and together with the down regulation of OPA-1 or Mfn, trigger alterations of the IMM structures which leads to fragmented mitochondria with greatly reduced oxygen consumption and electrochemical potential (Chen and Chan, 2005). Mitochondria also contribute to glycolysis regulation; in cases of increased ATP demand, mitochondrial pyruvate dehydrogenase activity is up regulated, increasing glycolytic contribution to cell metabolism (Sharma et al., 2005). The first evidence of a relationship between mitochondrial morphology and function derived from studies of fusion inhibition, which leads to a reduced oxygen consumption and loss of Δψmt and mitochondrial DNA (Chen et al., 2003; Chen and Chan, 2005). Silencing of Mfn-2 substantially impairs metabolic parameters (Pich et al., 2005), and animal models of obesity show a marked reduction of Mfn-2 levels in muscle cells. Similarly, decreased OPA-1 levels lead to mitochondrial fragmentation, decreased oxygen consumption, and Δψmt dissipation, and have been implicated in the pathogenesis of neurodegenerative diseases and heart failure (Bossy-Wetzel et al., 2003; Chen et al., 2009). Taken together, these results reinforce the concept that mitochondrial plasticity is critical for cell metabolism adaptation (Soubannier and Mcbride, 2009).

## Mitochondria–ER communication and metabolism

**Figure 2:**
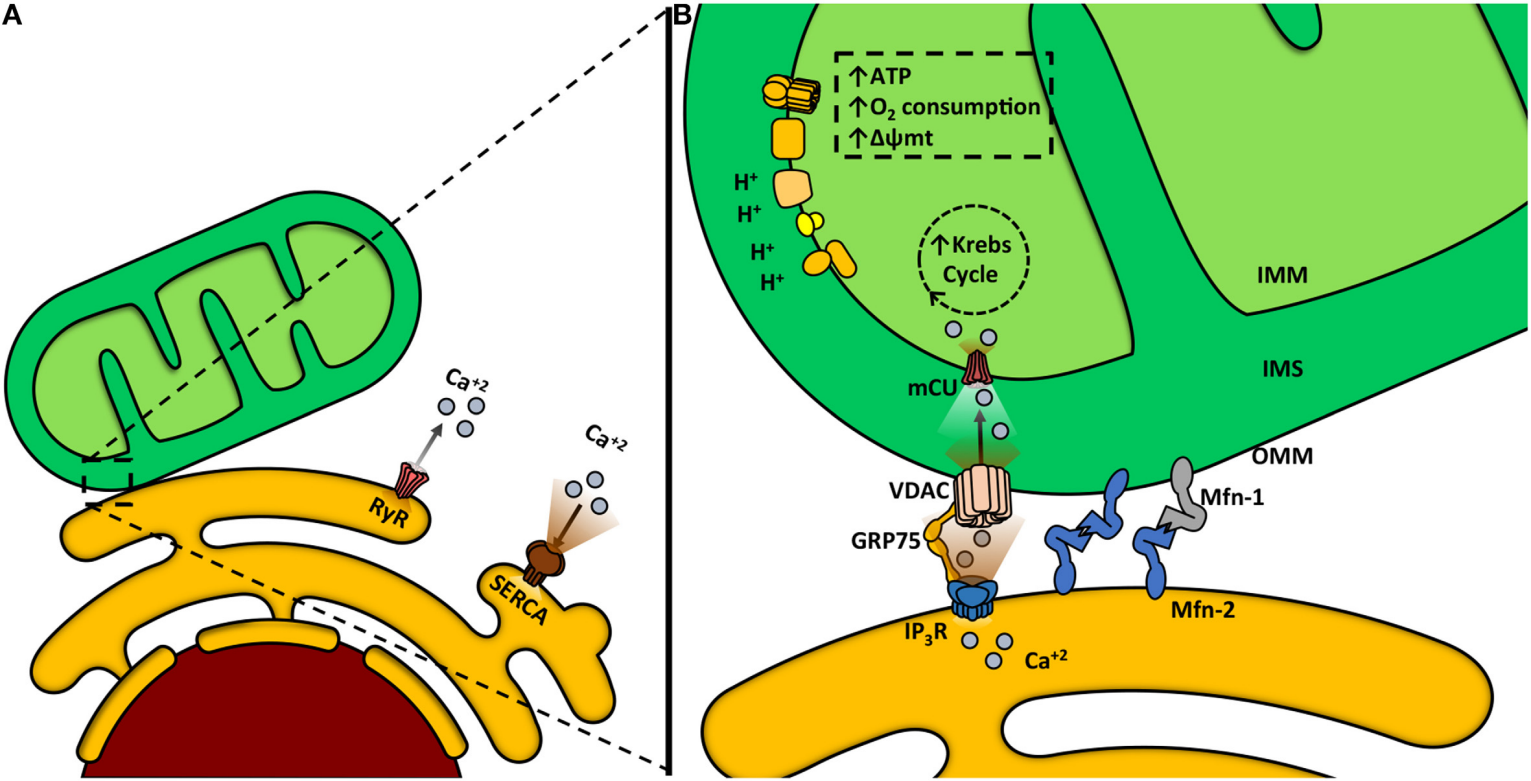
**Mitochondria-endoplasmic reticulum (ER) coupling**. **(A)** Ca^2+^ release from the ER to the cytosol is mediated by two channels: ryanodine receptor channel (RyR) and inositol trisphosphate receptor channel (InsP3R). The sarcoendoplasmic reticulum Ca^2+^ transport ATPase (SERCA) is a pump that transports Ca^2+^ from the cytoplasm into the ER. **(B)** Ca^2+^ transfer from the ER to the mitochondria occurs within the mitochondria-associated membranes (MAMs). Ca^2+^ is released from the ER to the mitochondria through the InsP3R. Glucose-regulated protein 75 (GRP75) is a mitochondrial chaperone that mediates the molecular interaction between InsP3R with the voltage-dependent anion channel (VDAC). Ca^2+^ import across the OMM occurs through VDAC. Ca^2+^ crosses the IMM through the mitochondrial Ca^2+^ uniporter channel (mCU) thanks to the considerable driving force represented by the negative transmembrane potential and the high Ca^2+^ concentration within the intermembrane space. Increased mitochondrial Ca^2+^ concentration activates mitochondrial dehydrogenases and enhances the oxidative phosphorylation, increasing oxygen consumption, Δψm and ATP production.

Like mitochondrion, ER is a highly dynamic organelle, undergoing constant shape remodeling, according to the cell requirements (Bravo-Sagua et al., 2013, 2014; English and Voeltz, 2013). Furthermore, both organelles also have great intracellular motility, being able to change their distribution in the subcellular space by moving along the cytoskeleton (Boldogh and Pon, 2007; Bola and Allan, 2009). This motility is important for cell physiology, because it determines the apposition between organelles and preferential sites of signaling. Among other functions, ER is the main intracellular Ca^2+^ reservoir. This ion is released to the cytoplasm by two channels, the ryanodine receptor channel (RyR) and the inositol trisphosphate receptor channel (InsP3R), and is returned to the ER by the activity of the sarco/endoplasmic reticulum Ca^2+^ ATPase (SERCA), a pump with high affinity and velocity (Figure 2A) (Berridge et al., 2003).

Ca^2+^ has several roles within the mitochondria, being required for protein synthesis (Joyal et al., 1995), activation of several citric acid cycle dehydrogenases, oxidative phosphorylation, ATP synthesis (Griffiths and Rutter, 2009), and it also enhances ROS production (Dykens, 1994). The OMM, initially considered a permeable barrier, plays a critical role in mitochondrial Ca^2+^ uptake (Csordas et al., 2002; Rapizzi et al., 2002). Ca^2+^ crosses this membrane through the low affinity channel VDAC, which is regulated by chaperones (Schwarzer et al., 2002), NADH (Lee et al., 1996) and Bcl-2 family proteins (Vander Heiden et al., 2001). Ca^2+^ then crosses the IMM through the mitochondrial Ca^2+^ uniporter (mCU), driven by the Δψmt (Kirichok et al., 2004; Baughman et al., 2011; De Stefani et al., 2011; Rizzuto et al., 2012). Ca^2+^ uptake into mitochondria requires high cytoplasmatic Ca^2+^ levels in the surrounding area, concentrations achieved in the proximity of Ca^2+^ release sites located in the ER such as the InsP3R (Figure 2B) (Csordas et al., 1999; Bravo-Sagua et al., 2013; Naon and Scorrano, 2014).

We and others have shown that functional coupling between these organelles promotes efficient Ca^2+^ entry into mitochondria, resulting in a higher Δψm and oxygen consumption (Figure 2B) (Cardenas et al., 2010; Bravo et al., 2011). Increased mitochondrial Ca^2+^ concentration enhances oxidative phosphorylation, through the activation of four mitochondrial dehydrogenases: FAD-glycerol 3-phosphate dehydrogenase, pyruvate dehydrogenase, NAD-isocitrate dehydrogenase and oxoglutarate dehydrogenase (Denton, 2009). In fact, blocking the entry of Ca^2+^ into the mitochondria reduces oxidative phosphorylation and energetically compromises the cell (Cardenas et al., 2010). Therefore, several dehydrogenases of the tricarboxylic acid cycle increase their activity in response to increments in mitochondrial Ca^2+^ content, which in turn depends on Ca^2+^ transients evoked by muscle contraction. This allows an appropriate coupling between contractile function and energy supply (Denton and Mccormack, 1990; Liu and O'Rourke, 2009).

**Figure 3:**
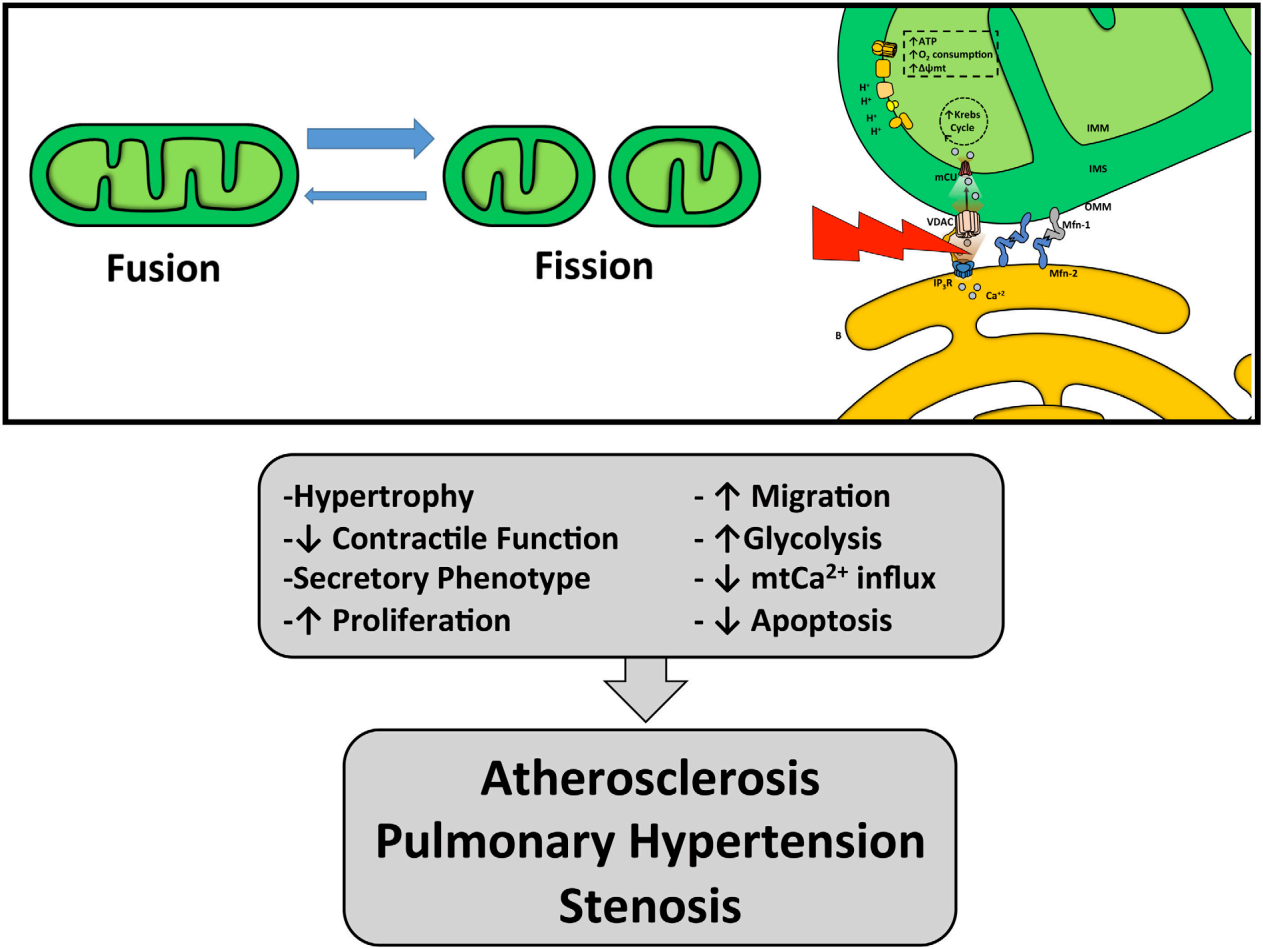
**Effects of mitochondria and ER–mitochondrial coupling dysfunction**. Unbalance in mitochondrial dynamics and ER–mitochondria uncoupling can lead to VSMC phenotypic switching promoting vascular pathologies such as atherosclerosis, stenosis and pulmonary hypertension.

The close contact zone between ER and mitochondria is called Mitochondria-Associated Membrane (MAM) (Vance, 2014). Several proteins compose and modulate this physical interaction, such as calcium channels (InsP3R, VDAC), mitochondria-shaping proteins (Mfn-2), chaperones (GRP75, sigma-1 receptor, calnexin, oxidoreductases), sorting proteins (PACS-2), and other enzymes [see reviews (Rowland and Voeltz, 2012; Bravo-Sagua et al., 2013, 2014)]. The physical junction between the ER and mitochondria relies on Mfn-2 and other proteins (De Brito and Scorrano, 2008). In fact, genetic ablation of Mfn-2 results in disrupted ER–mitochondria communication and causes a decrease in both Ca^2+^ transfer and mitochondrial bioenergetics (Figure 3) (Bravo et al., 2011; Bravo-Sagua et al., 2013).

Our group described changes in mitochondrial dynamics and mitochondrial metabolism associated to cardiomyocyte apoptosis (Parra et al., 2008), protection during heart ischemia/reperfusion (Zepeda et al., 2014), cardiomyocyte hypertrophy (Pennanen et al., 2014), and insulin resistance (Del Campo et al., 2014). We also showed that insulin regulates cardiomyocyte metabolism through the control of mitochondrial dynamics (Parra et al., 2014). We also described that the association between two organelles such as ER and mitochondria regulate mitochondrial energetic function, especially as a novel adaptative mechanism during ER stress (Bravo et al., 2011). Interestingly, when the association of both organelles is inhibited by a reduced expression of the protein Mfn2, the metabolic changes are abrogated (Bravo et al., 2011). On the other hand, it has been shown that ER–mitochondria coupling also can induce apoptosis through the connection of FIS-1 and Bap31 (an ER membrane protein). This association interacts as a scaffold complex which facilitates cell death signal transfer (Iwasawa et al., 2011).

## Mitochondria, VSMC proliferation, and apoptosis

Mitochondrial metabolism is being recognized as a critical regulator of cell growth and proliferation (Salabei and Hill, 2013). Cells in active division have enhanced glycolysis and glutamine utilization, which provide energy, NADPH, nucleotides, sugars, and amino acids required for cell proliferation (Moncada et al., 2012). In VSMCs, several growth factors, including PDGF-BB, increase mitochondrial activity (Moncada et al., 2012). In pulmonary artery hypertension (PAH) an increased proliferation rate of pulmonary artery smooth muscle cells (PASMCs) is observed. These cells have increased rates of glycolysis (Marsboom et al., 2012) and showed mitochondrial fission (Bonnet et al., 2006; Marsboom et al., 2012), suggesting a direct association between mitochondrial morphology and VSMC phenotypic change (Mitra, 2013). Salabei and Hill, proposed that the PDGF-BB-induced dedifferentiation of VSMCs occurs via two separate processes, i.e., proliferative responses driven by coupled transcriptional and bioenergetic processes, and loss of contractile proteins largely mediated by autophagy and downregulation of myofilament genes (Salabei and Hill, 2013). Moreover, inhibition of DRP-1 using Mdivi-1, triggers mitochondrial fusion and prevented PDGF-BB-induced upregulation of the cell cycle proteins proliferating cell nuclear antigen (PCNA) and cyclin D1 (Salabei and Hill, 2013).

VSMC proliferation and apoptosis can be also subject to mitochondrial control through the action Mfn-2. In spontaneously hypertensive or atherosclerosis-prone rat VSMC, Mfn-2 levels were diminished (Chen et al., 2004), while overexpression of Mfn2 reduced VSMC proliferation and sensitized to H_2_O_2_-induced apoptosis (Guo et al., 2007a,b). We have shown that the incretin glucagon-like peptide 1 (GLP-1) enhances the functional coupling between ER and mitochondria in VSMC and promotes a faster and higher increase of mitochondrial Ca^2+^ entry. This enhanced coupling was associated with an increase in mitochondrial metabolism because inhibition of Ca^2+^ release from ER, using the InsP3R blocker xestospongin B, or the blockade of Ca^2+^ entry to the mitochondria, using RuRed, abolished GLP-1-dependent increase of O_2_ consumption and Δψm (Morales et al., 2014). Therefore, it seems that communication between mitochondria and the ER is an efficient mechanism for promoting mitochondrial metabolism and regulating VSMC phenotype. This enhanced mitochondria–ER coupling is due to an increase of Mfn-2 through a protein kinase A (PKA)-dependent mechanism (Morales et al., 2014). Guo et al. observed that forskolin increases Mfn-2 expression in VSMC (Guo et al., 2007a). Similarly, treatment of mouse Leydig tumor cells with 8Br-cAMP also increases Mfn-2 mRNA and protein levels (Duarte et al., 2012). Moreover, Mfn-2 has potent metabolic effects. In type 2 diabetes patients, Mfn-2 mRNA and protein levels are diminished, and these levels correlate with increased body mass index and diminished insulin sensitivity (Bach et al., 2005; Hernandez-Alvarez et al., 2010). Furthermore, Mfn-2 depletion causes altered glucose metabolism *in vivo*, as Mfn-2-KO mice developed glucose intolerance, fasting hyperinsulinemia and altered insulin response (Figure 3) (Sebastian et al., 2012).

Chen et al. showed that Mfn-2 diminishes in highly proliferative VSMC from atherosclerosis-prone or balloon-injured rats and that Mfn-2 overexpression blocks proliferation of neointimal VSMC after balloon injury (Chen et al., 2004). Moreover, Mfn-2 overexpression also suppresses the proliferating effects of oxidized-LDL in rabbit VSMC cultures. The induction of Mfn-2 *in vivo* reduces PCNA positive cells at the neointimal and medial layers from rabbit carotid arteries subjected to air-drying damage (Guo et al., 2007b). These data correlate with the fact that overexpression of Mfn-2 promotes mitochondrial-mediated apoptosis in VSMC cultures, and that Mfn-2 is up-regulated and necessary for H_2_O_2_-induced apoptosis albeit in a mitochondrial fusion-independent mechanism (Guo et al., 2007a). This anti-proliferative activity of Mfn-2 can be negatively regulated by PKA as shown by the decreased PCNA positive cells and neointimal hyperplasia after balloon injury on rats with overexpression of a Mfn-2 S422A mutant form (a variant that cannot be phosphorylated on Ser422, the residue within the PKA-phosphorylation consensus site) (Zhou et al., 2010). The authors suggest that the anti-proliferative/pro-apoptotic activity of Mfn-2 might be a result of down-regulation of the Raf/MAPK pathway or control over apoptotic-related proteins, given that cells overexpressing Mfn-2 exhibit lower levels of ERK-1/2 and Akt in response to certain hormones (Chen et al., 2004; Guo et al., 2007a,b) and an increased Bcl-2/Bax ratio (Guo et al., 2007a).

During hypoxia most blood vessels relax but the pulmonary arteries constrict, ultimately becoming occluded by excessive PASMC proliferation, a condition that causes PAH. This is in part because during hypoxia, mitochondria from PASMCs modifies ROS production, which diffuse to the plasma membrane and regulate membrane voltage-dependent potassium channels (Kv) and activate the hypoxia induced factor (HIF-1α) signaling pathway, which is redox-sensitive (Michelakis et al., 2002). The inhibition of Kv channels by mitochondrial ROS cause depolarization, opening of voltage-gated Ca^2+^ channels, influx of Ca^2+^, and vasoconstrictive response (Dromparis et al., 2010). However, systemic VSMCs show totally opposite behavior of intracellular Ca^2+^ in response to hypoxia. Furthermore, there are many differences between PASMC and systemic VSMC mitochondria, such as decreased expression and function of the electron transporter chain complexes I–III and increased expression of superoxide dismutase (SOD). These differences are explained by differences in baseline of mitochondrial ROS between PASMCs and VSMCs, producing the opposite effect during hypoxia, contraction vs. dilatation, respectively (Michelakis et al., 2002).

During the incipient PAH, the mitochondria from PASMC become hyperpolarized and generate less ROS, indices of a metabolic shift. This event is produced during normoxia and can inhibit the Kv channel, which lead to an increase of intracellular Ca^2+^ levels and generate vessel contraction (Archer et al., 2008). In wild-type mice, these changes are accompanied by an excessive PASMC proliferation, decrease in glucose oxidation and increased glycolysis (Sutendra et al., 2010). When fatty acid oxidation is abolished by deletion of the malonyl-coenzyme A decarboxylase gene, thereby shifting the metabolic balance back to glucose oxidation, the mice do not develop PAH and less or no PASMC proliferation is observed (Sutendra et al., 2010). As mentioned above, the shift away from glucose oxidation toward glycolysis and fatty acid usage in the PASMCs during hypertension is accompanied by alterations in mitochondrial function (Sutendra et al., 2010). Intracellular Ca^2+^ level increase and glucose oxidation suppression activates transcriptional factors such as nuclear factor of activated T-cells (NFAT) which promotes PASMC proliferation in PAH (Bonnet et al., 2007; Sutendra et al., 2010). In PASMCs, HIF-1α activation induces cell proliferation and also triggers DRP-1-mediated mitochondrial fission. *In vivo*, cobalt activates HIF-1α and induces PAH in rats. Co-administration of Mdivi-1, restores mitochondrial fusion and reduces PAH (Marsboom et al., 2012). Increased fission observed in PASMCs from PAH results from DRP1 activation by increased cyclin B1/CDK1 activity and accompanies cell cycle progression from G2 to mitosis. DRP-1 inhibition by Mdivi-1 slows proliferation by locking mitochondria in fusion and inhibiting cell-cycle progression, causing G2/M arrest (Marsboom et al., 2012).

When ER stress is induced by chronic normobaric hypoxia in mice PASMCs, mitochondrial Ca^2+^, 2-oxoglutarate content and pyruvate dehydrogenase activity were decreased, depicting mitochondrial malfunction under these conditions (Sutendra et al., 2011). Sutendra et al. show that the Nogo-B protein, member of the reticulon family, which is critical in the regulation of tubular ER structure, is induced by hypoxia only in lung vessels, where it disrupts the contacts between the ER and the mitochondria (Sutendra et al., 2011). This alteration disrupts essential mitochondrial functions, causing overgrowth of PASMCs and ultimately PAH. Moreover, Nogo controls the ER shape and inhibits apoptosis during vascular remodeling (Sutendra et al., 2011).

## Mitochondrial dysfunction in atherosclerosis

Damage to the vascular environment by oxidative stress plays a major role in the pathogenesis of atherosclerosis. Under normal conditions, ROS production is controlled by mitochondria and ROS levels are sensed by enzymatic systems and cellular mechanisms. Alteration in ROS production is frequently observed in atherosclerosis. Oxidative stress results in lipid peroxidation and damage of mitochondrial components, including mitochondrial DNA (mtDNA), leading to mitochondrial dysfunction. mtDNA is also affected by mitochondrial fission process. Cleavage of OPA-1 leads to mtDNA loss, respiratory chain deficiency and mitochondrial ROS increase (Duvezin-Caubet et al., 2006; Finsterer, 2007; Parone et al., 2008). ROS increase activates poly(ADP-ribose) polymerase 1 (PARP-1), enzyme involved in chromatin structure modulation and DNA repair, which increases VSMC and endothelial cell death, facilitating atherosclerosis progression (Virag, 2005). Alteration in mitochondrial anti-oxidant enzyme levels, such as manganese superoxide dismutase (MnSOD), increases mtDNA damage in ApoE^−/−^ mice (Ballinger et al., 2002). Furthermore, the extent of mitochondrial, but not nuclear, DNA damage correlates with the development of atherosclerotic VSMC lesions in mice and human aortic tissues (Ballinger et al., 2002). Mitochondrial stress, induced by the mtDNA replication inhibitor, dideoxycytidine, promotes VSMC migration, but not proliferation, suggesting that malfunction of mitochondria may be involved in the plaque stabilization in late-stage atherosclerosis (Ahn et al., 2010). Plaque stabilization is accompanied by increase in VSMC and macrophage apoptosis which promotes induction of pro-coagulation and plaque rupture (Clarke et al., 2006). Also, VSMC functionality depends on mitochondrial genes transcription. VSMC-specific ablation of the mitochondrial transcription factor Tfam results in a diminished contractile response of mesenteric artery rings to phenylephrine (Jawien et al., 2008). Prevention of the upregulation of this transcription factor, observed in carotid artery from rats subjected to balloon injury, might prevent intimal thickening in this type of damaged-artery model (Yoshida et al., 2005). On the other hand, mitochondrial dysfunction in macrophages (changes in ATP production, Δψmt, and mtDNA damage) alters cholesterol trafficking between mitochondrial membranes facilitating cholesterol accumulation at the interface mitochondrial membrane–ER. This accumulation facilitates interaction between cholesterol and CYP27A1 due to the permeability transition pore (PTP) aperture, generating 27-oxygenated derivatives of cholesterol and accumulation within vascular cells (Allen et al., 2013). These results, among others, suggest that mitochondrial dysfunction is a possible hallmark of vascular diseases. Intervention and modulation of these mechanisms will be useful in the treatment and prevention of atherosclerosis progression.

## Concluding remarks

VSMC proliferation plays a key role in atherogenesis and restenosis. Mitochondrial dysfunction is a metabolic feature that control VSMC phenotype. Targeting of mitochondrial function with dichloroacetate (Mcmurtry et al., 2004) or trimetazidine (Sutendra et al., 2010) has been successfully used to avoid VSMC or PASMC proliferation in animal models. However, only rapamycin has been tested in human, specifically in drug eluting stents to avoid restenosis (Kang et al., 2011; Testa et al., 2011). Major translational challenges remain in this exciting area, but patients with vascular disease are likely to benefit from these efforts.

### Conflict of interest statement

The authors declare that the research was conducted in the absence of any commercial or financial relationships that could be construed as a potential conflict of interest.
